# The world health organization multicountry survey on maternal and newborn health: study protocol

**DOI:** 10.1186/1472-6963-11-286

**Published:** 2011-10-26

**Authors:** João Paulo Souza, Ahmet Metin Gülmezoglu, Guillermo Carroli, Pisake Lumbiganon, Zahida Qureshi

**Affiliations:** 1 UNDP/UNFPA/WHO/World Bank Special Programme of Research, Development and Research Training in Human Reproduction, WHO, Geneva, Switzerland; 2 Centro Rosarino de Estudios Perinatales, Rosario, Argentina; 3 Department of Obstetrics and Gynaecology, Faculty of Medicine, Khon Kaen University, Khon Kaen, Thailand; 4 Department of Obstetrics and Gynecology, University of Nairobi, Nairobi, Kenya; 5 on behalf of the WHO Multicountry Survey on Maternal and Newborn Health Research Group

## Abstract

**Background:**

Effective interventions to reduce mortality and morbidity in maternal and newborn health already exist. Information about quality and performance of care and the use of critical interventions are useful for shaping improvements in health care and strengthening the contribution of health systems towards the Millennium Development Goals 4 and 5. The near-miss concept and the criterion-based clinical audit are proposed as useful approaches for obtaining such information in maternal and newborn health care. This paper presents the methods of the World Health Organization Multicountry Study in Maternal and Newborn Health. The main objectives of this study are to determine the prevalence of maternal near-miss cases in a worldwide network of health facilities, evaluate the quality of care using the maternal near-miss concept and the criterion-based clinical audit, and develop the near-miss concept in neonatal health.

**Methods/Design:**

This is a large cross-sectional study being implemented in a worldwide network of health facilities. A total of 370 health facilities from 29 countries will take part in this study and produce nearly 275,000 observations. All women giving birth, all maternal near-miss cases regardless of the gestational age and delivery status and all maternal deaths during the study period comprise the study population. In each health facility, medical records of all eligible women will be reviewed during a data collection period that ranges from two to three months according to the annual number of deliveries.

**Discussion:**

Implementing the systematic identification of near-miss cases, mapping the use of critical evidence-based interventions and analysing the corresponding indicators are just the initial steps for using the maternal near-miss concept as a tool to improve maternal and newborn health. The findings of projects using approaches similar to those described in this manuscript will be a good starter for a more comprehensive dialogue with governments, professionals and civil societies, health systems or facilities for promoting best practices, improving quality of care and achieving better health for mothers and children.

## Background

Nearly 1,000 women die every day and ten million women present with complications related to pregnancy every year around the world [1]. Haemorrhage, infection, hypertensive disorders, obstructed labour and complications of unsafe abortion are the main pregnancy related complications that threaten women's life [2]. In addition, three million neonatal deaths, representing about 40% of all under-five infant mortality occur every year [3]. Three quarters of these neonatal deaths occur in the first week of life. Preterm birth, infections, and asphyxia are the major direct causes of neonatal deaths [4]. The vast majority of these deaths occur in developing countries and this burden motivated world leaders to formulate the millennium declaration, which has as outstanding targets the reduction of maternal and infant mortality in global scale. In September 2010, the United Nations Secretary-General launched the Global Strategy for Women's and Children's Health calling for concrete actions towards the improvement of health of women and children around the world [5].

Unfortunately, many of the complications leading to severe morbidity and deaths of mothers and newborns are not easily prevented. Several factors (individual, social, societal, health-system related etc) aggravate the vulnerability of mothers and children to complications and deaths related to pregnancy and childbirth. However, timely and optimal treatment can largely improve survival [6,7]. The evidence shows that high maternal, perinatal, neonatal and child mortality rates are associated with inadequate and poor quality health services. Evidence also suggests that explicit, evidence-based, cost effective packages of interventions can improve the processes and outcomes of health care when appropriately implemented. The interventions for life support and emergency obstetric care include the administration of parenteral antibiotics, uterotonic drugs, anticonvulsants, manual removal of placenta, removal of retained products of conception, assisted vaginal delivery, obstetric surgery (caesarean section and hysterectomy), safe blood transfusion, resuscitation of the newborn and corticosteroids during preterm labour which can reduce maternal and newborn mortality [6,8]. Complications not recognized in a timely manner or not treated appropriately are likely to progress to organ dysfunction and deaths. Furthermore, even despite appropriate initial care, some women and newborn may develop organ dysfunctions which constitute a common final pathway towards death [9,10]. At this stage, more specialized and expensive interventions would be necessary to revert life-threatening conditions related to pregnancy and childbirth. In this continuum, the timing and appropriateness of care can explain part of the huge difference observed between developed and developing countries in terms of maternal and infant mortality.

Auditing deaths is an approach commonly used for assessing the quality of care and identifying opportunities for improvement [11]. Women who survive life-threatening conditions arising from complications related to pregnancy and childbirth have many common aspects with those who die of such complications. This similarity led to the development of the near-miss concept in maternal health. Exploring the similarities, the differences and the relationship between women who died and those who survived life-threatening conditions provide a more complete assessment of quality in maternal health care [12-15].

### The WHO approach to the near-miss concept in maternal and newborn health

A systematic review on the prevalence of severe acute maternal morbidity and maternal near-miss was conducted by the WHO in 2004 [12]. This systematic review found a wide variation of criteria used to identify near-miss cases. Owing to the variations in the identification criteria, the corresponding severity of the "near-miss" cases identified by different authors was too heterogeneous and a summary estimate for maternal near-miss prevalence was not feasible. In 2007, WHO established a technical working group of obstetricians, midwives, epidemiologists and public health professionals from developing and developed countries to produce a standard definition and uniform identification criteria for maternal near-miss cases. Aiming to achieve a reasonable balance between the burden of data collection and useful information, this working group targeted the identification of very severe cases, essentially those presenting with features of organ dysfunctions. A standard definition has been developed, tested and validated. Detailed information about the near-miss concept and its development is published elsewhere [16]. The maternal near-miss concept was suggested to be routinely used in national programmes as tool for evaluating the quality of maternal health care [17].

In addition, indicators attached to the near-miss concept were developed for the assessment of quality of care. The near-miss indicators are indicators of outcome and provide an overall evaluation of the performance of the health service or health system in reducing severe maternal short-term outcomes [16]. In order to produce a more complete and even more tangible evaluation of quality of care, a set of process indicators was adapted and developed based on the criterion-based clinical audit (CBCA) concept. CBCA is considered a feasible and beneficial mode of auditing the quality of maternal health care [18]. These process indicators assess the use of priority effective interventions in the prevention and management of severe complications related to pregnancy and childbirth and can be tailored according to local standards of care.

Similar to the maternal near-miss approach, neonates that nearly died but survived severe complications at birth or during the neonatal period (e.g. infants who survived extreme preterm birth, very low birth weight, birth asphyxia, birth trauma, neonatal sepsis) could be studied as surrogates of neonatal deaths [19]. There is currently no standard definition for neonatal near miss. In this context, a newborn that requires a life-saving intervention (e.g. intubation) and did not receive it will very likely die. Thus, life-saving interventions could be an entry point to initiate the development of the neonatal near-miss definition, together with other indicators of increased risk of death [20]. Near-miss indicators, similar to those established in the maternal near-miss context, could be used to explore this new field and assess the quality of care provided to newborns with severe complications [19,20].

In summary, effective interventions to reduce mortality and morbidity in maternal and newborn health already exist and many of these interventions should be put in practice at health facilities [6,7]. Information about quality and performance of care and the use of critical interventions would be useful for shaping improvements in health care and strengthening the contribution of health systems towards the Millennium Development Goals 4 and 5 [21]. The near-miss concept and the criterion-based clinical audit are proposed as useful approaches for obtaining such information in maternal and newborn health care.

## Objectives

In this paper, we present the methods of the World Health Organization Multicountry Survey on Maternal and Newborn Health. The main objectives of this study are to determine the prevalence of maternal near-miss cases in a worldwide network of health facilities, evaluate the quality of care using the maternal near-miss concept and the criterion-based clinical audit, and explore the use of the near-miss concept in perinatal health. As this study follows the WHO Global Survey on Maternal and Perinatal Health and maintains key methodological features of that study, we also highlight some of their differences.

## Methods/Design

### Research design and cluster selection

This is a cross-sectional study which will be implemented in the network of health facilities that participated in the previous WHO Global Survey on Maternal and Perinatal Health. These facilities were identified through a multi-stage sampling method [22]. The first stage of sampling was the selection of countries. This selection was stratified according to the WHO regions and the levels of under-five child and adult mortality. Fourteen sub-regions constituted the sampling frame for the first stage of selection. From each sub-region, a total of four countries were selected at random for participation in the study, with probability proportional to the country population. When the total number of countries was less than four in any sub-region, all the countries were included. This process resulted in 12 sub-regions having four countries each and two sub-regions having three countries each. A total of 54 countries were initially pre-selected, but, due to operational and budgetary reasons, the WHO Global Survey project was implemented in 24 countries from Africa, Asia and Latin America. The second stage of sampling consisted of random selection of two provinces/states (with probability also proportional to the population size), in addition to the capital city in these 24 countries. A third sampling stage (also based on the population size but reaching sub-divisions below the province/state level) was used for very large provinces/states. For very large cities (e.g. Mexico City and Beijing), a fourth sampling stage was implemented based on the random selection of city geographical sub-divisions, with probability proportional to city sub-division population. Once the geographical areas were selected, seven health facilities with a minimum of 1,000 deliveries per year were randomly selected from each of these areas with probability proportional to the annual number of deliveries. If there were less than 7 facilities, all facilities in that area were selected. In the end, the WHO Global Survey Project showed that such a sampling scheme was feasible and could represent the facility-based health care systems available in the countries (private, social service, public, etc) [22]. For the present study, the existing network of health facilities has undergone adjustments considering logistic factors, resource issues, and the availability and motivation of collaborating facilities. Afghanistan and Pakistan, countries that were previously selected to participate in the Global Survey project, have been included in the multicountry survey and the selection of health facilities has followed the previously used random sampling scheme. Mongolian health facilities were also added to the network using the same procedures. In addition, selected facilities from Jordan, Lebanon, Palestine (West Bank) and Qatar were added to the network. The health facilities from Cuba, Algeria and the Mexican State of Tamaulipas were discontinued. In the end, 370 health facilities from 29 countries (i.e. Afghanistan, Angola, Argentina, Brazil, Cambodia, China, Democratic Republic of the Congo, Ecuador, India, Japan, Jordan, Kenya, Lebanon, Mexico, Mongolia, Nepal, Nicaragua, Niger, Nigeria, Pakistan, Palestine, Paraguay, Peru, Philippines, Qatar, Sri Lanka, Thailand, Uganda, Viet Nam) will participate in this study.

### Study participants

During the data collection period, each health facility will include all eligible participants. The eligible participants are:

• all women giving birth during the data collection period in the participating hospitals together with their respective newborns;

• all maternal near-miss cases admitted in the participating hospitals up to seven postpartum/postabortion days, regardless of the gestational age and delivery status;

• all maternal deaths taking place in the participating hospitals up to seven postpartum/postabortion days, regardless of the gestational age and delivery status.

The WHO criteria will be used to identify maternal near-miss cases (Table 1). Participants will be excluded if they were admitted to the participating hospitals after seven days of termination of pregnancy (delivery or abortion).

**Table 1:** The WHO maternal near-miss criteria*

	Identification criteria
**Cardiovascular dysfunction**	• Shock
	• Use of continuous vasoactive drugs
	• Cardiac arrest
	• Cardio-pulmonary resuscitation
	• Severe hypoperfusion (lactate > 5 mmol/L or > 45 mg/dL)
	• Severe acidosis (pH < 7.1)
	
**Respiratory dysfunction**	• Acute cyanosis
	• Gasping
	• Severe tachypnea (respiratory rate > 40 bpm)
	• Severe bradypnea (respiratory rate < 6 bpm)
	• Severe hypoxemia (PAO2/FiO2 < 200 or O2 saturation < 90% for ≥60 min)
	• Intubation and ventilation not related to anaesthesia
	
**Renal dysfunction**	• Oliguria non responsive to fluids/diuretics
	• Dialysis for acute renal failure
	• Severe acute azotemia (creatinine ≥ 300 umol/ml or ≥ 3.5 mg/dL)
	
**Coagulation/hematologic dysfunction**	• Failure to form clots
	• Massive transfusion of blood or red cells (≥ 5 units)
	• Severe acute thrombocytopenia (< 50,000 platelets/ml)
	
**Hepatic dysfunction**	• Jaundice in the presence of pre-eclampsia
	• Severe acute hyperbilirubinemia (bilirubin > 100 umol/L or > 6.0 mg/dL)
	
**Neurologic dysfunction**	• Prolonged unconsciousness (lasting ≥ 12 hours)/coma (including metabolic coma)
	• Stroke
	• Status epilepticus/uncontrollable fits
	• Total paralysis
	
**Uterine dysfunction**	• Haemorrhage or infection leading to hysterectomy

* Detailed information available at references [16] and [24]

### Data collection and data management

This study will collect data at two levels, the individual and the facility level. At the individual level, the study participants (and their respective newborns) will have their medical records reviewed, whereas at the facility level, data will be collected through a specific survey among the professionals responsible for the participating facilities.

Study variables are described in detail in the study protocol and manual of operations. In brief, at the individual level, these variables include maternal and newborn individual data, data related to the pregnancy outcomes, severe complications and their management. At the facility level, the characteristics of each health facility and their ability to identify and manage severe complications will be investigated.

At the individual level, in each health facility, data collectors will perform daily visits to the obstetrical/postpartum ward, gynaecologic/abortion care unit, delivery room and intensive care unit to identify women with life-threatening conditions. Upon discharge from hospital or in the event of maternal death, the study participants will have their medical records reviewed. The facility medical staff will be questioned for doubts during data collection or missing information.

In general, the duration of data collection will be two months if the health facility had 6,000 deliveries/year or more (in the year that preceded the study implementation) and three months if the health facility had less than 6,000 deliveries/year. In Jordan, Lebanon, Palestine (West Bank) and Qatar, the data collection period will be extended to four months in all health facilities considering the number and characteristics of health facilities in order to obtain meaningful data. There will be no individual follow-up of women or newborns after hospital discharge. Data will be collected only from the hospital medical records with regards to the intra-hospital care up to the seventh day following delivery or abortion. With this sampling scheme, the expected sample size will be around 275,000 women.

The individual level and the cluster level data will be initially collected on paper forms. Then, data will be entered onto a web-based data management system developed by the Centro Rosarino de Estudios Perinatales (CREP, Rosario, Argentina). This software was designed according to the main standards defined by the United States Food and Drug Administration [23]. The WHO Coordinating Unit and the centres will decide whether online data entry will take place at the facility or at a more central level depending on the resources available in each centre. Regional data managers will monitor the data flow and its quality using data validation and progress reports that are automatically generated by the web-based system. These procedures have been used in previous multicentre studies, including the WHO Global Survey on Maternal and Perinatal Health [22].

### Quality control procedures

The majority of the facilities have participated in the previous WHO Global Survey on Maternal and Newborn Health and the data collectors and their supervisors are conversant with the data collection and entry methods. Training will be emphasized in those facilities that are new in the network. Online data entry system will minimize the data entry errors and facilitate monitoring and quick resolution of queries and missing data. Data inconsistencies will be identified and corrected as they occur, using specific reports included in the web-based data management system and parallel arrangements, in order to obtain a clean database soon after the end of data collection in each site. A manual of operations has been developed to minimize the need for judgement and interpretation by the data collectors. The manual of operations includes a description of the study in general terms, emphasizes the importance of complete and accurate data, and fosters the standardization of data collection. The data collection tools have been reviewed by other researchers and pre-tested on a convenient sample of records and clinical settings. Training workshops at country and facility levels will be carried out and tailored according to specific needs. In each country, a pilot phase will be implemented in order to test the complete data management process (data collection, data entry and query procedures). The total number of women delivering at the facilities during data collection will be independently monitored and these numbers will be compared to those determined by the data collection. Intra-form validity cross-checks will be performed in addition to random cross-checks comparing medical records against form and electronic data.

The staff responsible for data will maintain a log book to document unanticipated problems. Technical questions encountered in the field will be resolved through consultation with the country and regional coordinators under the supervision of the WHO coordinating unit. Missing data in medical records will be obtained from the attending physician.

### Ethical considerations

This is an observational study, in which data will be collected and extracted from the health facility medical records without any identification of the study participant. Data will be extracted anonymously from hospital records with no personal identifiers and reported cumulatively. Information will not be obtained directly from the study participants, nor will participant interviews take place. The facility medical staff may be asked to clarify doubtful or missing information during data collection. Information on the participating subject (i.e. name, individual identification code at the hospital, birth date and delivery date) will be kept in the log book at the institutional level (by a person in charge of data collection) to help completeness of the form in case significant details are missed from being recorded or if the queries are raised at the data cleaning stage. Data collectors and other study staff will ensure the confidentiality of logbooks and other data storage devices (e.g. computers) by ensuring that logbooks are not taken out of the hospital premises, keeping them in locked lockers, password secured databases and secure removal of computer data/shredding of any study logbooks at completion of central database cleaning. Therefore, individual informed consent from the individual study participants is not necessary.

Data collectors will be selected among the health facility staff. If additional study staff is employed (i.e. not a staff of that specific health facility) to extract data from facility records, the additional staff will sign a confidentiality agreement and report to the facility management and the study investigators. Thus, the additional study staff will be governed by the same rules in relation to confidentiality and legal indemnity that will also govern the conduct of hospital staff. In any case, as a facility based study, an authorization to perform the study will be obtained at the institutional level from the responsible authority (director or medical chief) in all selected health facilities. At any country or health facility in which this study is to be implemented the relevant ethical clearance should be obtained. This study protocol has been approved by the World Health Organization Ethical Review Committee.

### Project implementation and management

This project is a complex operation involving many people at facility, country, continental and global levels. The overall coordination of the project is carried out by the global coordinating unit at the WHO Department of Reproductive Health and Research in Geneva, Switzerland. There are regional coordinators for Africa, Americas and Asia. The regional coordination for Afghanistan and Pakistan will be provided by the regional coordinator of Asia. Each country will have a country coordinator. The country coordinator will be responsible for coordinating data collection from all the selected health facilities within that country. Due to practical reasons, coordination for Jordan, Palestine and Qatar will be provided by the Lebanon country coordinator, who will liaise directly with the global coordination. There will be one coordinator from each selected health facility. The health facility coordinator will be assisted by a data collector/s who will be responsible for the day to day collection of the data from the records and a data clerk/s who will assist in online data entry. Despite being one single project involving 29 countries, in practice, this project functions as a coordinated set of 29 studies using the same research protocol in 29 countries. Data collection started in May 2010 and will be finalized in all countries by the end of 2011.

### Analysis plan

At country level and for every country, a descriptive analysis will be carried out with emphasis on the maternal near-miss indicators and the criterion-based clinical audit process indicators.

At the global level, two main analyses are anticipated, one focused on maternal health, using the maternal near-miss approach, and other focused on newborn health. The resulting estimates will always refer to the facility-based sample. Analysis techniques for a stratified multi-level sampling design will be used to obtain descriptive data including worldwide, regional, and country estimates of prevalence of severe maternal complications (i.e. causes and other conditions associated to maternal deaths and maternal near-miss cases), maternal near miss, and neonatal conditions. The associations between the use of interventions and the maternal and perinatal outcome will be examined. The proportions between maternal complications, maternal near miss and maternal deaths will be used to assess the quality of care. All analyses will consider the influence of potential confounding variables. Multilevel modelling will be used, which offers the flexibility of simultaneously controlling for confounding variables while estimating the prevalence of outcomes and the assessment of interaction. Multiple logistic regressions adjusted for clustering will be explored.

The general analytical approach for the main objectives follows:

• The prevalence of maternal near miss will be determined. The overall estimates with 95% confidence interval will be calculated. The proportions of women with maternal complications, maternal near miss and maternal deaths will be assessed.

• Descriptive frequencies of the use of interventions will be calculated (e.g. use of prophylactic and therapeutic uterotonics, use of parenteral anticonvulsants for eclampsia, ICU admission for life-threatening conditions etc). The relationship between the use of these interventions and the maternal and perinatal outcome will be evaluated by calculating odds ratio (crude and adjusted), with 95% confidence intervals.

• A set of process indicators have been developed according to pre-defined algorithms. Essentially, we will evaluate the proportion of women who did receive a specific intervention compared to the population that should have received the specific intervention (for instance, the total number of women with eclampsia that received magnesium sulphate compared to the total number of women with eclampsia). This approach may indicate gaps in the implementation of evidence-based practices. Country and overall estimates will be calculated. This information will be related to the best estimate of effect available for each selected intervention and the avoidable burden of the complication will be estimated.

• Markers of severity will be studied as potential criteria for identifying neonatal near-miss cases (Table 2). Neonatal near-miss indicators, similar to those established in the maternal near-miss context, will also be used to explore this objective. Individual and cluster level analyses are expected, evaluating, for instance, the relationship between availability of life-saving interventions and the neonatal outcome.

**Table 2:** Conditions to be tested as criteria for identifying neonatal near-miss cases.

	Identification criteria
**Clinical organ dysfunction**	• Respiratory rate > 100 breaths per minute
	• Cyanosis in room air
	• Absence of regular breathing pattern (gasping respiration or frequent apnoea)
	• Cardiac arrest
	• Persistent bradycardia < 80 bpm
	• Persistent tachycardia > 200 bpm
	• Poor capillary filling (> 5s)
	• Subaponeurotic haemorrhage
	• Seizures
	• Severe neurological depression (inability to suck)
	• Severe pallor
	• Visible jaundice in first 24 hours
	• Any active, non traumatic, bleeding (e.g. GI bleeding, pulmonary haemorrhage)
	• Visible haematuria
	• Anuria > 24 hours
	• Apathetic/Poor tolerance of feeds
	• Abdominal distension and vomiting
	• Brachial plexus injury
	• Skull fracture
	
**Laboratory markers of organ dysfunction**	• Saturation by pulse oximetry < 85% in room air
	• pCO2 > 65 mmHg
	• Serum pH < 7.1
	• Haematocrit < 30%
	• Haemoglobin < 10 g/dl
	• Glucose < 30 mg/dl or < 1.7 mmol/l
	• White cell count < 4000 cells/mm3
	• Neutropaenia < 1000 cells/mm3
	• Raised C-Reactive Protein within 48 hours > 10 mg/dlx.
	• X-ray signs of intestinal obstruction/perforation
	• X-ray signs of skull fracture
	
**Management indicators of severity**	• Any intubation (at birth or anytime within first week)
	• Nasal CPAP
	• Ventilation
	• Surfactant administration
	• Cardio-pulmonary resuscitation (cardiac massage)
	• Use of any vasoactive drug
	• Volume expansion
	• Use of anticonvulsants
	• Use of phototherapy in the first 24 hours
	• Exchange transfusion
	• Use of any blood products
	• Use of steroids to treat refractory hypoglycaemia
	• Use of therapeutic IV antibiotics
	• Any surgery requiring general anaesthesia
	
**Other conditions highly associated with severity in perinatal health**	• Birth weight < 1500 g
	• Gestational age at birth < 31 weeks
	• Apgar score 5 minutes < 5

Several other secondary analysis exploring different aspects of the global database are expected, including comparisons with the previous WHO Global Survey on Maternal and Perinatal Health database.

## Discussion

This paper outlines the study protocol for a large World Health Organization multinational study using the maternal near-miss concept and a criterion-based clinical audit approach. This protocol has been developed through a consultative process involving a large number of stakeholders from several countries. In addition to the WHO study, a number of other studies are implementing this protocol with minor adaptations. Combining the expected number of participants in our study with those of other initiatives that we are aware of, over one million women, from at least 800 health facilities around the world will be included in studies using research protocols based on the original protocol outlined in this paper. Thus, this study protocol will be the basis for obtaining important evidence about the use of the near-miss concept in maternal and newborn health. In addition to the information on the prevalence of complications, it will ascertain whether markers of severe maternal morbidity can be incorporated into routine data collection systems, and provide a standardized evaluation of quality of care in a large number of health facilities in different geographical regions. In the end, this study will provide a comprehensive evaluation of the implementation status of critical life-saving interventions in the continuum of maternal and perinatal care.

However, the implementation of this study protocol may face some challenges. The first one is that the maternal near-miss concept is relatively new and the criteria used to identify maternal near-miss cases are based on organ dysfunction markers. These markers are not part of the traditional, routinely collected information in maternal health. Raising awareness among the health care professionals who work in the participating health facilities and motivating them to contribute to the systematic identification of near-miss cases are essential components of projects based on this protocol. Another relevant issue is the fact that this study is facility-based. Our network is mostly composed of medium and large facilities, many of them functioning as referral hospitals in their geographical areas. This may introduce a bias, overestimating the prevalence of severe complications. The results will be applicable to facility-based settings, a fact especially relevant for countries with low health-facility coverage. Given the data collection challenges involved in conducting a large multi-hospital, multi-country study, and, in order to keep the data collection burden to a minimum, we have chosen to measure only short-term, intra-hospital maternal and perinatal morbidity and mortality. Thus, medium and long-term maternal and perinatal outcomes of potentially serious consequence are not covered by this study.

In order to further facilitate the use of the methods of the WHO Multicountry Survey on Maternal and Newborn Health, we developed a study protocol derivative that complements the previously published papers about the maternal near-miss definition and criteria. The generic guide for implementing the WHO near-miss approach in maternal health services has been prepared as a simplified version of this protocol and discusses alternatives to some of the methods used in our study [24].

Investigators using this study protocol or its derivatives are encouraged to report and publish their findings. We anticipate conducting a systematic review and meta-analysis of studies that used the WHO criteria for maternal near miss or developing a repository to accommodate those studies and results. Thus, we propose that authors of studies using the WHO maternal near-miss criteria report a minimum set of information. This would include a description of the setting where the near-miss approach was implemented, the study eligibility criteria, the period of data collection, the procedure for case identification, crude value of variables necessary to calculate the near-miss indicators (i.e. the total number of live births in the source population, the number of maternal deaths and the number of near-miss cases), and an interpretation of the findings considering the local context. We also suggest that the term "maternal near miss" is included as a key word for convenient indexation and facilitation of retrieval of publications for future systematic review and meta-analyses.

## Conclusion

Implementing the systematic identification of near-miss cases, mapping the use of evidence-based critical interventions and analysing the corresponding indicators are just the initial steps for using the maternal near-miss concept as a tool to improve maternal and newborn health. Tailored multifaceted approaches, possibly including the use of evidence-based guidelines and reminders, engagement of opinion leaders, and continued audit and feedback may be needed in selected areas [24,25]. Ultimately findings of projects using approaches similar to those described in this paper need to be put in action. These findings will be a good starter for a more comprehensive dialogue with policy makers, professional and civil societies, health systems or health services administrators for promoting best practices, improving quality of care and achieving better health for mothers and children.

## Competing interests

The authors declare that they have no competing interests.

## Authors' contributions

This project is the result of an international collaborative effort carried out by a large group of institutions and researchers members of the World Health Organization Multicountry Survey on Maternal and Newborn Health Research Group (WHOMCS Research Group). JPS prepared the original study protocol in collaboration with AMG. Substantial input to the study protocol was provided by the study regional coordinators (GC, PL, ZQ) and the WHOMCS Research Group. This manuscript was drafted by JPS on behalf of the WHOMCS Research Group with contributions from AMG, PL, GC, PL, ZQ. Members of the WHOMCS research group (particularly LS, ML, BF, BH and SM) provided substantive input to this manuscript. All members of the WHOMCS Research Group read and approved the final manuscript.

## Pre-publication history

The pre-publication history for this paper can be accessed here:

http://www.biomedcentral.com/1472-6963/11/286/prepub
